# 15-oxoeicosatetraenoic acid mediates monocyte adhesion to endothelial cell

**DOI:** 10.1186/s12944-017-0518-2

**Published:** 2017-07-12

**Authors:** Guohua Ma, Bing Pan, Sufen Ren, Caixia Guo, Yansong Guo, Lixin Wei, Lemin Zheng, Buxing Chen

**Affiliations:** 1 Department of Cardiology, Beijing Tian Tan Hospital, Capital Medical University, Beijing, 100050 China; 2 The Institute of Cardiovascular Sciences and Institute of Systems Biomedicine, School of Basic Medical Sciences, and Key Laboratory of Molecular Cardiovascular Sciences of Ministry of Education, Peking University Health Science Center, Beijing, 100191 China; 3 Tai Zhou Municipal Hospital, Taizhou, 318000 China; 4 Department of Cardiovascular Medicine, Fujian Provincial Hospital, Fuzhou, China; 5 Department of Nephrology, Union Hospital, Fujian Medical University Union Hospital, Fuzhou, China

**Keywords:** 15-oxo-ETE, monocyte adhesion, E-selectin, Atherosclerosis, PKC

## Abstract

**Background:**

A great number of studies reported that 12/15-lipoxygenase (12/15-LO) played an important role in atherosclerosis. And its arachidonic acid(AA) metabolite, 15(S)-hydroperoxy-5,8,11,13-(Z,Z,Z,E)-eicosatetraenoic acid (15(S)-HETE), is demonstrated to mediate endothelial dysfunction. 15-oxo-5,8,11,13-(Z,Z,Z,E)-eicosatetraenoic acid (15-oxo-ETE) was formed from 15-hydroxyprostaglandin dehydrogenase (PGDH)-mediated oxidation of 15(S)-HETE. However, relatively little is known about the biological effects of 15-oxo-ETE in cardiovascular disease. Here, we explore the likely role of 15-lipoxygenase (LO)-1-mediated AA metabolism,15-oxo-ETE, in the early pathogenesis of atherosclerosis.

**Methods:**

The 15-oxo-ETE level in serum was detected by means of liquid chromatography and online tandem mass spectrometry (LC-MS/MS). And the underlying mechanisms were illuminated by molecular techniques, including immunoblotting, MTT assay, immunocytochemistry and *Immunohistochemistry.*

**Results:**

Increased 15-oxo-ETE level is found in in patients with acute myocardial infarction (AMI). After 15-oxo-ETE treatment, Human umbilical vein endothelial cells (HUVECs) showed more attractive to monocytes, whereas monocyte adhesion is suppressed when treated with PKC inhibitor. In ex vivo study, exposure of arteries from C57 mice and ApoE−/−mice to 15-oxo-ETE led to significantly increased E-selectin expression and monocyte adhesion.

**Conclusions:**

This is the first report that 15-oxo-ETE promotes early pathological process of atherosclerosis by accelerating E-selectin expression and monocyte adhesion. 15-oxo-ETE -induced monocyte adhesion is partly attributable to activation of PKC.

**Electronic supplementary material:**

The online version of this article (doi:10.1186/s12944-017-0518-2) contains supplementary material, which is available to authorized users.

## Background

Arachidonic acid (AA) is a key polyunsaturated fatty acid (PUFA), which derives a great number of bioactive mediators that are involved in many physiological and pathological processes. AA can be oxygenated by a variety of different enzymes, including lipoxygenases, cyclooxygenases, and cytochrome P450s [1], this study will focus on the biosynthesis of 15-oxo-5,8,11,13-(Z,Z,Z,E)-eicosatetraenoic acid (15-oxo-ETE) in 15-LOX pathway. Two types of 15-LOX have been identified in human, including 15-LOX-1 (encoded by ALOX15 gene) and 15-LOX-2(encoded by ALOX15B gene) [2, 3]. In humans, 15-LOX is expressed in various tissue cells and plays different physiopathologic function [2, 4]. Notably, 15-LOX is highly expressed in macrophages within human atherosclerotic plaques [5–7]. In macrophages, 15-LOX-1 converts AA to 15-hydroperoxyeicosatetraenoic acids (15-HpETEs), which is soon reduced by peroxidase to 15-hydroxyeicosatetraenoic acids (15-HETEs). Finally, 15-HETEs was oxidized to 15-oxoETE by 15-hydroxyprostaglandin dehydrogenase (15-PGDH), which requires NAD+ as a cofactor.

There is substantial evidence for the pro-atherosclerotic effect of 15-LOX: (i) downregulation of 12/15-LOX reduced lipid oxidation and foam cell formation [8]; (ii) up-regulation of 15-LOX-2 in macrophages enhances inflammation and the recruitment of inflammatory cells in hypoxic atherosclerotic plaque [6]; (iii) 15-LOX is a key enzyme for the oxidization of low-density lipoprotein (LDL) [9–11]; (iv) disruption of 12/15-LOX gene decreased high-fat diet induced endothelial dysfunction and vascular inflammation [12]; (v) ALOX15B is highly expressed in carotid lesions in patients with cerebrovascular symptoms. 15-HETE, as the initial 15-LOX metabolite of AA, was first identified in human atherosclerotic plaque using high-performance liquid chromatography [13]. It is reported that 15S–HETE may be involved in endothelial dysfunction, monocyte/macrophage transmigration [12], vascular wall remodeling [14], and angiogenesis [15]. Moreover, a recent research showed that 15-HETE promoted oxidative stress and foam cell formation [16]. However, the evidence for the role of 15-oxoETE in atherosclerosis is unclear.

A large body of evidence suggests that endothelial dysfunction is a key variable in the pathogenesis of atherosclerosis [17–19]. Endothelial dysfunction, as a comprehensive index of the overall CVD risk factor burden includes three main consequences, exposure of adhesion molecules, the activation and aggregation of platelets, cholesterol accumulation [20]. Monocyte recruitment is the initial incident in atherosclerotic plaque formation, the present study was designed to explore the likely effect of 15-oxo-ETE on monocyte recruitment.

## Methods

### Study population

We enrolled patients with diagnosis of acute myocardial infarction (AMI) (*n* = 8) and patients without coronary artery lesion admitted for elective coronary diagnostic (*n* = 8). In addition, all the subjects didn’t take aspirin or other NSAIDs until first medical contact in Beijing TianTan hospital.

### Measurement of AA metabolites

Blood samples for 15-oxoETE assays were drawn into EDTA tubes from a radial artery approach before coronary angiography. After centrifugation, blood samples were stored at −80 °C until AA derivative measurement. Plasma AA derivative levels were quantified by ultra-high performance liquid chromatography-tandem mass spectrometric (UPLC-MS/MS). Briefly, Waters Oasis-HLB cartridges were washed with 1 ml methanol and 1 ml MilliQ water, and samples with internal standard mixture (each 5 ng) were loaded into the cartridges and washed with 5% methanol (1 ml). The aqueous plug was then pulled from the SPE cartridges under high vacuum for 20 min, and the analytes were eluted into tubes with 1 ml methanol. Samples were dried using a Thermo Speed Vac, dissolved in 120 μl 30% ACN, and filtered with a 0.22 μC for UPLC-MS/MS. A total of 31 AA mediators were detected.

### 15-oxoETE and animals

15-oxoETE was purchased from Cayman Chemical (Ann Arbor, MI). C57 mice (6–8-week-old males) and ApoE−/− mice (6–8-week-old males) were obtained from the animal house of Peking University, Beijing, China.

### Cell lines and cell culture

The human monocyte cell line THP-1 was purchased from Cell Resource Center, Institute of Basic Medical Sciences, Chinese Academy of Medical Sciences, Beijing, China. Cells were cultured in RPMI1640medium (Hyclone, USA) containing 10% fetal bovine serum. HUVECs were isolated from umbilical veins [21]. The cells were cultured in ECM (ScienCell, USA) containing 5% FBS, 1% endothelial cell growth supplement, and 1% penicillin/streptomycin solution. The cells were then cultured in a humidified incubator of 5% CO_2_ at 37 °C. HUVECs were used at passages 2–5.

### 3-(4,5-Dimethylthiazol-2-yl)-2,5-diphenyltetrazolium bromide (MTT) assay

For cell viability assay, HUVECs (5 × 10^3^ cells/well) were seeded in 96-well plates and cultured overnight, subsequently treated with varying concentrations of 15-oxoETE (0, 2.5, 5, 10, 20, 40 μmol/L) for 24 h or 15-oxoETE (0, 0.5, 1, 2, 4, 8 μmol/L) for 12 h. And then added 10 μl MTT solution to each well at a final concentration of 0.5 mg/ml and incubated for an additional 4 h. At the end of incubation, dimethylsulfoxide (150 μl) was added to each well and then the absorbance was measured at 570 nm using an enzyme-linked immunosorbent assay (ELISA) reader.

### Measurement of adhesion molecules expression in HUVECs

Adhesion molecules expression was measured by immunocytochemistry. Briefly, HUVECs were plated in 96-well plates. Confluent HUVECs were serum starved for 6 h and treated with 15-oxoETE (0, 0.5, 1, 2, 4, 8 μmol/L) for 6 h at 37 °C, TNF-α was served as positive control. Then HUVECs were incubated with Rabbit Anti-ICAM-1 (PB0053, Boster) or Rabbit Anti -VCAM-1 (BA0406, Boster) or Rabbit Anti -E-selectin (BA0615, Boster) (1:200; Boster, China) in First Antibody Dilution Buffer for 2 h 37 °C. Omission of primary antibody was conducted in Negative controls. And then cells were incubated with a horseradish peroxidase-conjugated antibody (1:1000, Abcam, USA) for 1.5 h at 37 °C. Quantification was performed by measuring the absorbance at 450 nm by a TMB peroxidase EIA substrate kit (Bio-Med Innovation, China) [22].

### In vitro adhesion assay

In brief, confluent HUVECs on 96-well plates were starved for 6 h and treated with 15-oxoETE (0, 0.5, 1, 2, 4, 8 μmol/L) for 6 h at 37 °C. Thereafter, cells were exposed to human THP-1 monocytes (10^5^cells/well) for 40 min. Non-adherent THP-1 cells were removed by washing one time with PBS, the absorbance of adherent cells at 450 nm was measured. Six parallel wells were set up for each group. Adherent cells were counted in randomly selected optical fields taken from an inverted microscope (Leica). PKC inhibitor, staurosporine (2.5 nmol/L), was used at the beginning of treatment [23].

### PKC activity assay

The PKC activity in the HUVEC lysates was determined by PepTag® Non-Radioactive Protein Kinase Assays (Promega, V5330, USA). As described in the manufacturer’s instructions, 5 × 10^6^cells were removed to prepare PKC extracts. The brightly colored, fluorescent peptide substrate was specially phosphorylated by PKC, and its net charge was altered from +1 to −1. This change in the net charge of the substrate allowed the phosphorylated and non-phosphorylated versions of the substrate to be rapidly separated on an agarose gel [22].

### Immunoblotting

Bicinchoninic acidprotein assay kit was purchased from Thermo Fisher Scientific (USA). E-selectin (ab18981) and secondary antibodies was purchased from Cell Signaling Technology (USA). Details of immunoblotting have been described elsewhere [24].

### Immunohistochemistry

Histology tissues were fixed in 4% paraformaldehyde and subsequently embedded in paraffin wax. 6-μm-thick sections were cut from each paraffin-embedded tissue and collected on microscope slides, and then dewaxed with xylene and rehydrated with ddH_2_O. Endogenous peroxidases were blocked by 0.3% H_2_O_2_ solution (dilute with methanol) for 12 min. Antigen retrieval was conducted in EDTA working solution (10 mmol/L, pH 8.0) for 10 min at 92 °C. The tissue sections were blocked by 5% goat serum for 1.5 h at 37 °C, after which sections were incubated with primary antibodies (rabbit anti-ICAM-1 (PB0054, Boster), VCAM-1 (BA0406, Boster), or E-selectin (BA0615, Boster); dilution 1:200) overnight at 4 °C. The tissue sections were then treated with appropriate HRP-conjugated secondary antibodies for 40 min at 37 °C, and Antigen-antibody reactions were stained with 3, 3-diaminobenzidine. The adhesion molecules expression was examined with a Nikon Eclipse Ti microscope under high power (400×) fields. Adhesion molecules expression was quantified by Leica Q550CW image analysis system.

### Ex vivo adhesion assay

For ex vivo adhesion experiments, 150 μl PBS and 150 μl 15-oxoETE (314 μM) were respectively given by tail vein injection. 24 h later, mice were anaesthetized with ketamine and 5% chloral hydrate. Under sterile conditions, the heart was exposed through a left thoracotomy, and perfused from the left ventricle with normal saline until the blood was washed out. Then Hoechst-labeled THP-1 monocytes (1 × 10^7^cells/ml) were perfused from the left ventricle to the aorta and incubated for 45 min at 37 °C. Non-adherent THP-1 cells were removed by perfusion with normal saline. Aortas were harvested at the level of the aortic arch to the abdominal aorta beyond renal arteries and subsequently fixed in 4% paraformaldehyde solution. Adhesion was observed by confocal laser scanning microscope and quantified by calculating the areas of fluorescent monocytes attached to the vascular surface.

### Statistical analysis

Statistical analysis was carried out using one way ANOVA with Dunnett’s post-test and Student *t* test; *p* < 0.05 was considered significant. Error bars represent mean ± SD.

## Result

### Patient clinical characteristics

A total of 16 patients were included in the study. Among enrolled patients, 8 patients presented with stable angina pectoris, 4 patients with non ST-segment elevated MI and 4 patients with ST-elevated MI. The coronaries of patients in control group are all normal or close to normal. Patients in AMI group all have one an infarcted-related artery with 80%-100 stenosis. Patient clinical characteristics are summarized in Table 1. The details of patients are provided in Additional file 1.

**Table 1:** The clinical characteristics of patients are summarized as follow

Variable	Control	AMI
Age (years) (mean ± SD)	56.4 ± 8.5	60.0 ± 8.8
Male sex (male/total)	7 (87.5)	7(87.5)
Hyperlipidemia (n, %)	7 (87.5)	7(87.5)
Diabetes mellitus (n)	0	0
Hypertension (n, %)	4 (50)	6(75)
Smoking (n, %)	7(87.5)	7(87.5)
History of ischemic heart disease (n)	0	0
Clinical presentation		
SAP(n, %)	8(100)	0(0)
Non-STEMI(n, %)	0(0)	4(50)
STEMI(n, %)	0(0)	4(50)

### AA metabolites of two groups

**Fig. 1:**
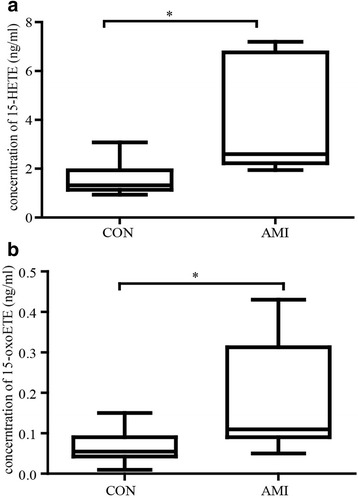
Quantification of 15-HETE and 15-oxoETE. The levels of 15-HETE (**a**) and 15-oxoETE (**b**) were compared between AMI and control groups. (*P < 0.05)

We found that several AA metabolite components showed difference between control and AMI groups (Table 2). Among CYP450 pathway metabolites, 11,12-DHET (*P* < 0.05), 14,15-DHET (*P* < 0.01), 16-HETE (*P* < 0.05), 18-HETE (*P* < 0.01), 20-HETE (*P* < 0.01), 5,6-DHET (*P* < 0.01) and 8,9-DHET (*P* < 0.05) levels were significant higher in AMI group compared with control group. However, no difference is found in COX_6 k–PGF1a while other metabolites in COX pathway were undetectable. Interestingly, among 15-LOX pathway, 15-HETE and 15-oxoETE are both higher in AMI group than control group (*P* < 0.05) (Fig. 1), indicating their likely pro-atherosclerosis role in AMI.

**Table 2:** A total of 31 AA mediators were detected by UPLC-MS/MS, “-” means that the compound was measured but was undetectable

		Control (*n* = 8)	AMI (*n* = 8)	P
	AA*	104,946 ± 50,057	199,020 ± 104,954	0.0382
COX	6 k–PGF1a	3.4780 ± 1.8930	3.3490 ± 2.1540	0.9008
	15d–PGJ2	-	-	
	PGB2	-	-	
	PGD2	-	-	
	PGE2	-	-	
	PGF2a	-	-	
	PGJ2	-	-	
	TXB2	-	-	
LOX	11-HETE*	0.4650 ± 0.3027	1.020 ± 0.5969	0.0343
	12-HETE	4.2260 ± 2.5060	11.2100 ± 10.5400	0.0896
	15-HETE*	1.5640 ± 0.7041	4.0450 ± 2.368	0.0131
	15-oxo-ETE*	0.0650 ± 0.0424	0.1788 ± 0.1391	0.0441
	5-HETE*	0.8913 ± 0.4127	1.6000 ± 0.0672	0.0233
	5-oxo-ETE	0.9000 ± 0.0298	0.1525 ± 0.159	0.2944
	8-HETE**	0.4438 ± 0.2850	0.9000 ± 0.3100	0.0084
	9-HETE*	0.3688 ± 0.2658	0.8425 ± 0.4803	0.0285
	LTB4	0.0243 ± 0.0229	0.0425 ± 0.0385	0.2950
CYP450	11,12-DHET*	0.1888 ± 0.0564	0.2975 ± 0.0932	0.0135
	11,12-EET	0.1988 ± 0.1133	0.2400 ± 0.0919	0.4374
	14,15-DHET**	0.4013 ± 0.1015	0.6450 ± 0.1706	0.0037
	14,15-EET	0.2188 ± 0.1228	0.3425 ± 0.1276	0.0681
	17-HETE	-	-	
	19-HETE	-	-	
	16-HETE*	0.0750 ± 0.0093	0.1386 ± 0.0684	0.0214
	18-HETE**	0.1250 ± 0.0367	0.2338 ± 0.0902	0.0070
	20-HETE**	0.5312 ± 0.1997	1.3840 ± 0.6527	0.0031
	5,6-DHET**	0.0875 ± 0.0315	0.1850 ± 0.0731	0.0038
	5,6-EET	0.6525 ± 0.4443	0.7238 ± 0.4517	0.7551
	8,9-DHET*	0.1000 ± 0.0293	0.1438 ± 0.0403	0.0263
	8,9-EET	0.1700 ± 0.0894	0.2325 ± 0.0836	0.1708

*P < 0.05, **P < 0.01 (unit:ng/ml)

### Cell viability of HUVECs undergoing 15-oxoETE treatment

**Fig. 2:**
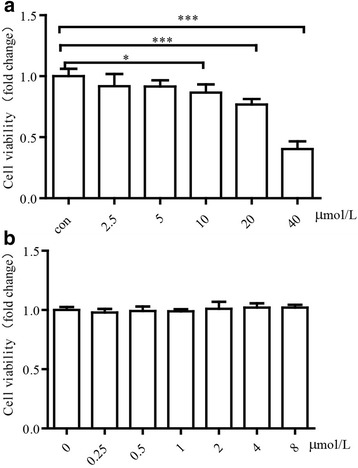
The effect of 15-oxoETE on cell viability. HUVECs were exposed to different 15-oxoETE levels 15-oxoETE (0, 2.5, 5, 10, 20, 40 μmol/L) for 24 h (**a**) and 15-oxoETE (0, 0.5, 1, 2, 4, 8 μmol/L) for 12 h (**b**), cell toxicity assay was tested by MTT assay. Results are presented as the mean ± SD of three independent experiments. (*P < 0.05, ***P < 0.001)

To explore the influence of 15-oxoETE on cell viability, MTT assay were conducted on HUVECs treated with varying doses (0-40 μmol/L) of 15-oxoETE for a 24 h period. The cells exhibited a dose-dependent reduction in viability (Fig. 2a). Endogenous 15-oxoETE is so low that it can’t kill cells in short period. Therefore, lower doses of 15-oxoETE (0-8 μmol/L) were added to cultured HUVECs under serum deprivation, and then the cell viability was assessed by MTT assay after 12 h. HUVECs showed no change when treated with concentrations of 0-8 μmol/L, which will be applied to the subsequent experiments (Fig. 2b).

### 15-oxo-ETE promotes monocyte adhesion and positively regulates E-selectin expression

**Fig. 3:**
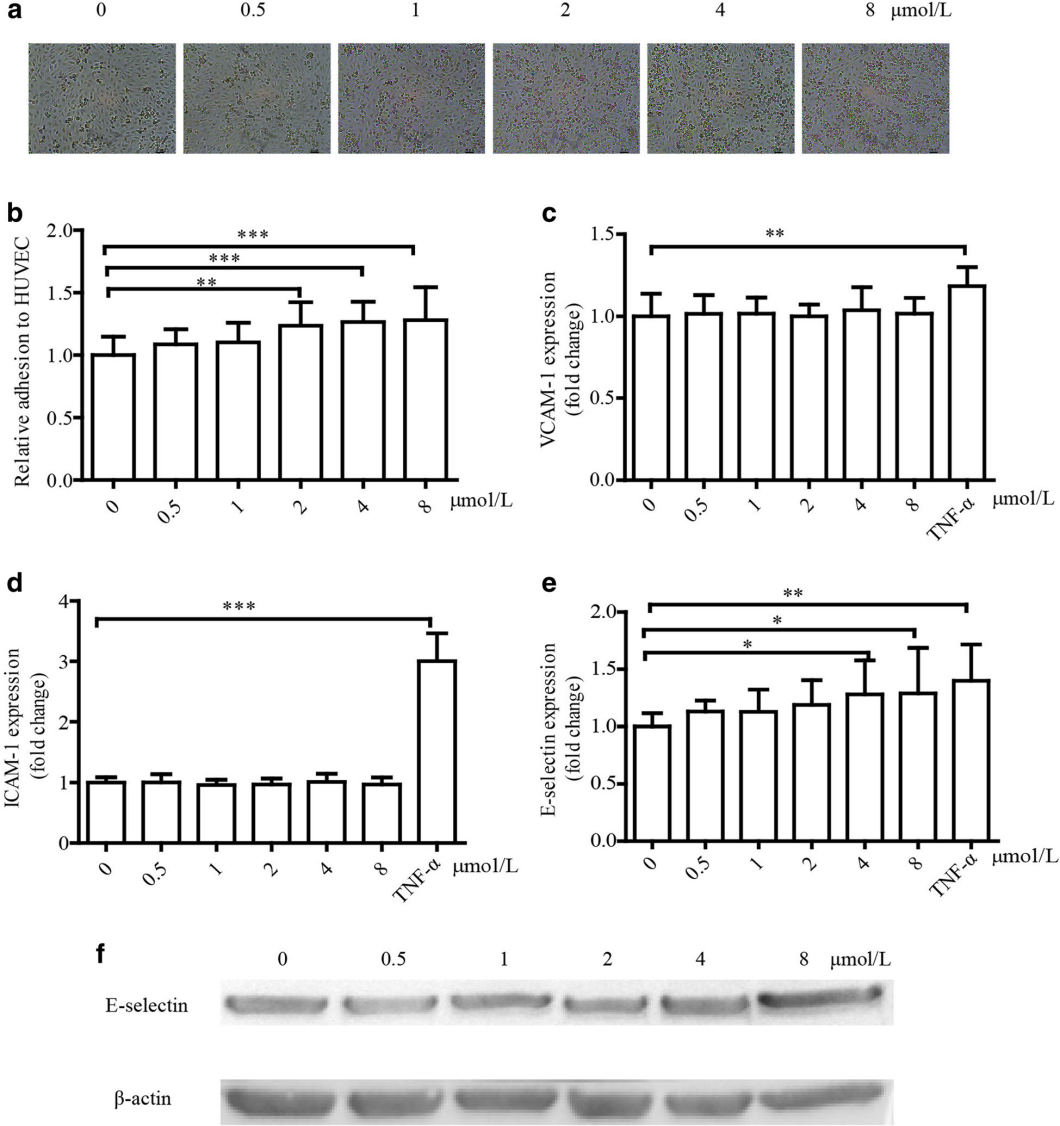
15-oxoETE treatment promotes monocyte recruitment and increases E-selectin expression in vitro. Monocyte recruitment was illustrated by panels A and B. **a** HUVECs were starved for 6 h and treated with 15-oxoETE (0, 0.5, 1, 2, 4, 8 μmol/L) for 6 h. Images represent the mean of adherent monocytes per microscopic field. Scale bar = 50 μm. **b** Monocyte adhesion was quantified by manual count, The results were normalized to the number of control cells. **c**, **d**, **e** HUVECs were treated with 15-oxoETE at the indicated concentrations as above for 6 h. VCAM-1, ICAM-1, and E-selectin expression in HUVECs were examined by cell ELISA. Results are represented as fold of 15-oxoETE-treated cells in comparison with control, TNF-α was served as positive control. **f** Effect of 15-oxoETE on the protein level of E-selectin was determined by western blot. All data are expressed as mean ± SD of three independent experiments. (*P < 0.05, **P < 0.01)

Monocyte adhesion is a vital process in the pathogenesis of atherosclerosis. To uncover the effect of 15-oxo-ETE on monocyte adhesion to HUVECs, HUVECs were treated with various doses of 15-oxo-ETE for 6 h and co-cultured with THP-1 cells for an additional 40 min. Adhesion of THP-1 cells to HUVECs was stimulated by 15-oxo-ETE in a dose-dependent manner as the amount of adherent THP-1 cells increased from 1.00 ± 0.15 to 1.28 ± 0.26 (Fig. 3a, b).

During the process of cell adhesion, cell adhesion molecules most likely play an important role in cell communication [25–27]. The cell ELISA results suggested that treatment of 15-oxo-ETE up-regulated E-selectin, but not ICAM-1 and VCAM-1 (Fig. 3c, d). When HUVECs were treated with 8 μmol/L of 15-oxo-ETE, expression of E-selectin reached 1.40 ± 0.32 fold increase compared with control (Fig. 3e). The protein level of E-selectin was also up-regulated as analyzed by Western blot (Fig. 3f).

### 15-oxo-ETE promotes THP-1 adhesion to mice aortic wall and increases E-selectin expression in mice aortic ECs

**Fig. 4:**
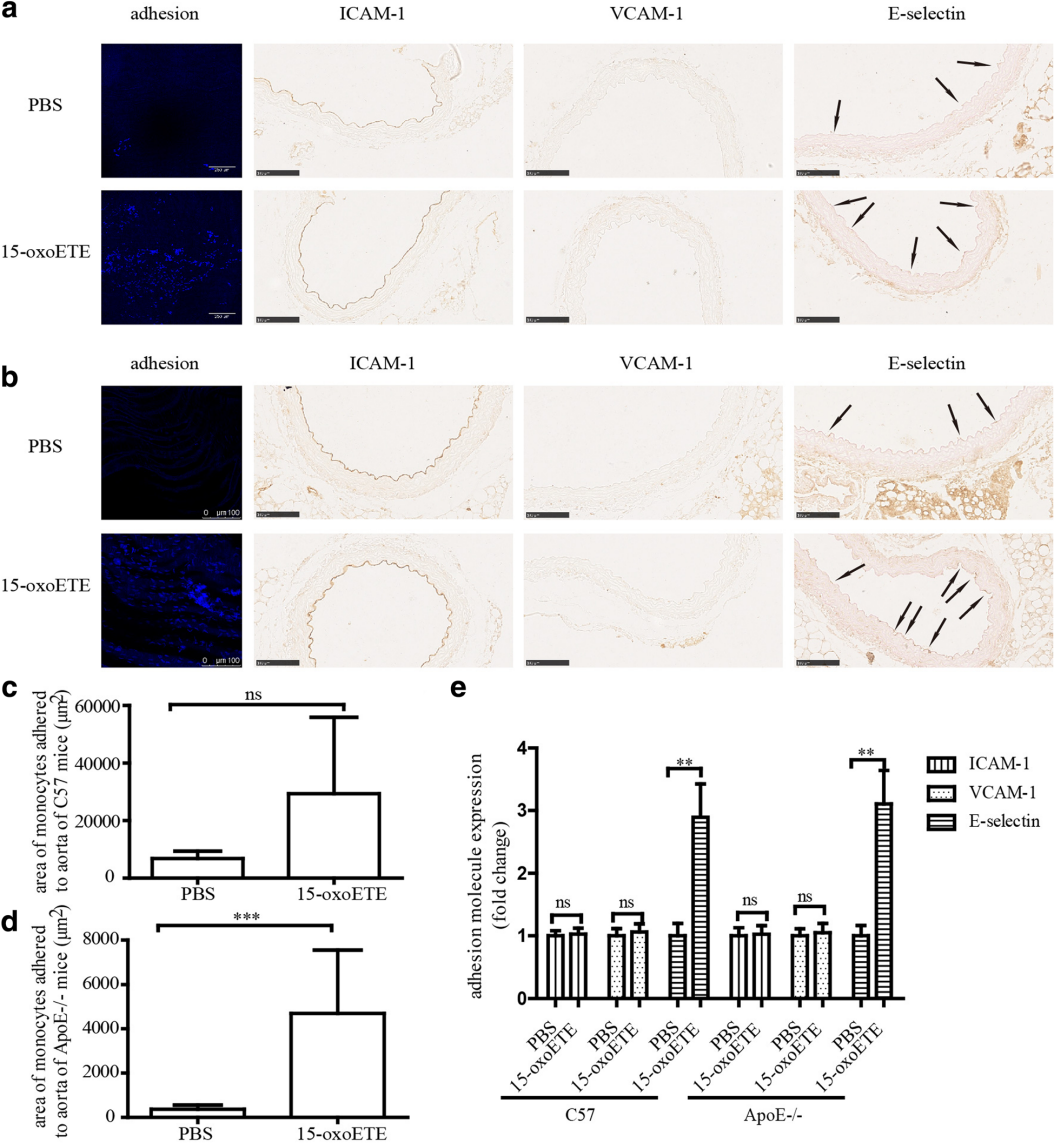
15-oxoETE injection enhances monocyte adhesion and E-selectin expression ex vivo. **a** 15-oxoETE treatment increased THP-1 cell adhesion to the aorta vascular wall of C57 mice. Results were shown as fluorescence in confocal microscopic images. Scale bars = 250 μm. Subsequent images represent the immunohistochemical staining of ICAM-1, VCAM-1, and E-selectin in vascular ECs. Arrows represent E-selectin-positive areas. Scale bars = 50 μm, (*n* = 3). **b** 15-oxoETE treatment increased THP-1 cell adhesion to the aorta vascular wall of ApoE−/− mice. Results were shown as fluorescence in confocal microscopic images. Scale bars 100 μm. Subsequent images represent the immunohistochemical staining of ICAM-1, VCAM-1, and E-selectin in vascular ECs. Arrows represent E-selectin-positive areas. Scale bars = 50 μm, (*n* = 3). **c**, **d** Quantification of adhesion on aorta, the result was expressed as areas of monocytes attached to aorta. Data are expressed as mean ± SD. **e** Quantification of adhesion molecules expression, the fold change is presented as mean ± SD. (**P < 0.01, ***P < 0.001)

To further confirm the contribution of 15-oxo-ETE-induced monocyte adhesion ex vivo, we performed adhesion assays with aorta isolated from C57 mice and ApoE−/− mice. Treatment with 15-oxo-ETE make an increase in monocytes adhesion to the aorta of C57 mice, but no significant difference is showed (4.29 ± 3.90 fold change, *P* = 0.1376) (Fig. 4a, c) and E-selectin expression (2.89 ± 1.93 fold change) (Fig. 4e). Coincidently, exposure of arteries from ApoE−/−mice to 15-oxo-ETE led to significantly increased monocyte adhesion (12.46 ± 7.55 fold change, *P* < 0.05) (Fig. 4a, c) and E-selectin expression (3.11 ± 1.78 fold change) (Fig. 4e). However, no change was observed in the expression of ICAM-1and VCAM-1 compared with control (Fig. 4e).

### PKC activation is required for 15-oxoETE-induced monocyte adhesion

**Fig. 5:**
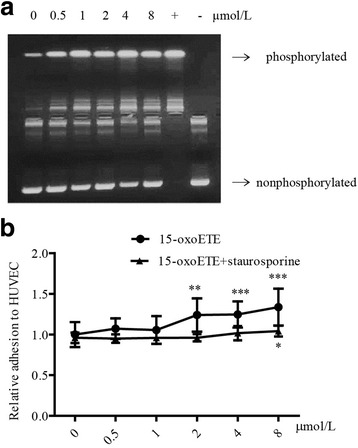
15-OXO-ETE-induced monocyte adhesion is attributed to PKC activation. **a** HUVECs were treated with 15-oxoETE (0, 0.5, 1, 2, 4, 8 μmol/L) for 6 h in serum-free media. Cell lysates from 15-oxoETE-treated cells were analyzed by the PepTag nonradioactive PKC assay. **b** Monocyte adhesion was measured in the presence or absence of staurosporine (2.5 nmol/L), the results are presented as the mean ± SD of three independent experiments (*P < 0.05, **P < 0.01, ***P < 0.001, compared with control)

It has been known that the activity of PKC plays an important role in regulating endothelial dysfunction, including inflammation and adhesion [23]. PKC activity was examined in HUVECs treated with 15-oxoETE. In the present study, 15-oxoETE accelerated PKC phosphorylation compared with control in a dose-dependent manner (Fig. 5a). Interestingly, staurosporine treatment significantly diminished the increased monocytes adhesion in every concentration level of 15-oxoETE, especially in higher concentrations of 2–8 μmol/L (Fig. 5b). These data indicated that the activation of PKC was responsible for 15-oxoETE-induced monocytes recruitment.

## Discussion

15-oxoETE is firstly found in rabbit lung, which is produced from 15-PGDH-mediated oxidation of 15-HETE [28]. Cong Wei et al. found that 15-oxoETE inhibited endothelial proliferation in a dose-dependent manner [29]. There was also a study suggested that 15-oxoETE promoted carotid artery construction during hypoxia [30]. These results indicated that 15-oxoETE might play an important role in atherosclerosis. Conversely, 15-oxoETE is found to protect pulmonary arterial smooth muscle cells against serum deprivation-induced apoptosis, indicating a potential therapeutical role of 15-oxoETE in pulmonary arterial hypertension [31]. What’s more, 15-oxoETE was reported to mediate pro-inflammatory signaling by activating anti-inflammatory Nrf2 signaling and down-regulating pro-inflammatory cytokine [32]. Taken together, this interesting metabolite of AA remains a biological curiosity in atherosclerotic pathophysiology. Thus, this study attempts to demonstrate that 15-oxoETE promote pathological process of atherosclerosis by accelerating monocytes recruitment.

More recently, 15-oxo-ETE was identified as a metabolite of AA in primary human monocytes with IL-4 treatment. They indicated that 15-oxoETE had a short half-life of only 11 min, and then crossed the cell membrane rapidly [29]. It may be the reason why people found 15-HETE but not 15-oxoETE is the main AA metabolite in atherosclerotic plaque. Interestingly, the present UPLC/MS/MS analysis showed that both 15-HETE and 15-oxoETE level is elevated in AMI patients (Table 2, Fig. 1). Thus, we hypothesized 15-oxoETE was released from macrophagocytes in atherosclerotic plaque to affect vascular function.

Vascular inflammation and monocyte adhesion are critical events in the initiation of atherosclerosis [25, 33]. The increased expression of cellular adhesion molecules, such as VCAM-1 E-selectin and ICAM-1, plays an important role in endothelial inflammation and is essential to recruiting monocytes from the circulation [33, 34]. The present study suggested that 15-oxoETE positively regulated E-selectin expression in HUVECs and increased monocyte adhesion to HUVECs (Fig. 3). Consistently, the result is further tested in both C57 and ApoE−/− mice. What’s more, the adhesion change is more significant in ApoE−/− mice than C57 mice (Fig. 4). These data revealed that 15-oxoETE might be a risk factor of cardiovascular disease in both health groups and high-risk groups.

PKC activation strengthens the adhesion reaction of endothelial cells with monocytes [35, 36]. Four PKC isoforms have been identified in human ECs, namely PKC-α, PKC-δ, PKC-ε, and PKC-ζ [27]. Although the present study did not explore the roles of different PKC isoforms in 15-OXO-ETE-treated ECs, we showed that total PKCs activity was increased in 15-oxoETE-treated ECs (Fig. 5a). Moreover, increased monocyte adhesion was significantly diminished by PKC inhibitor (Fig. 5b). Therefore, we speculate that PKC activation may play an important role in 15-oxoETE induced monocyte adhesion.

## Conclusion

These studies provide a novel role of 15-oxoETE in atherosclerosis. 15-oxoETE promotes E-selectin expression and PKC-dependent monocyte adhesion, which indicating that 15-oxoETE is probably a potential risk factor of atherosclerosis. This question will be better clarified until abundant convictive clinical data will be given.

## Additional file

Additional file 1: The details of patients are provided as follow. (DOCX 32 kb)

## Abbreviations

15(S)-HETE: 15(S)-hydroperoxy-5,8,11,13-(Z,Z,Z,E)-eicosatetraenoic acid
15-HpETEs: 15-hydroperoxyeicosatetraenoic acids
15-oxo-ETE: 15-oxo-5,8,11,13-(Z,Z,Z,E)-eicosatetraenoic acid
AA: Arachidonic acid
AMI: Acute myocardial infarction
HUVECs: Human umbilical vein endothelial cells
LAD: Left anterior descending
LC-MS/MS: Liquid chromatography and online tandem mass spectrometry
LCX: Left circumflex artery
LDL: Low-density lipoprotein
Non-STEMI: Non ST-segment elevated myocardial infarction
PGDH: 15-hydroxyprostaglandin dehydrogenase
PUFA: Polyunsaturated fatty acid
RCA: Right coronary artery
SAP: Stable angina pectoris
STEMI: ST-segment elevated myocardial infarction
