# Supplementary material for: 15-oxoeicosatetraenoic acid mediates monocyte adhesion to endothelial cell
Source: Lipids Health Dis. 2017 Jul 12;16:137. doi: 10.1186/s12944-017-0518-2 (PMC5508789; doi:10.1186/s12944-017-0518-2)
Supplement: Supplementary file 1 [file 12944_2017_518_MOESM1_ESM.docx]

control									
ID	gender	age	Dyslipidemia	Hypertension	Smoking	History of MI	Diabetes mellitus	Clinical presentation	main lesion
481552	male	68	yes	yes	yes	no	no	stable angina pectoris	normal
482026	male	49	yes	no	yes	no	no	stable angina pectoris	normal
482641	male	43	yes	yes	yes	no	no	stable angina pectoris	normal
482881	male	59	yes	no	yes	no	no	stable angina pectoris	normal
440215	male	57	yes	yes	yes	no	no	stable angina pectoris	normal
440154	male	66	yes	no	yes	no	no	stable angina pectoris	almost normal
492946	male	58	yes	yes	yes	no	no	stable angina pectoris	normal
467538	female	51	no	no	no	no	no	stable angina pectoris	normal
AMI group									
ID	gebder	age	Dyslipidemia	Hypertension	Smoking	History of MI	Diabetes mellitus	Clinical presentation	main lesion
468295	female	73	yes	yes	yes	no	no	Non-STEMI	RCA(90% stenosis)
467006	male	63	yes	yes	yes	no	no	Non-STEMI	LAD(90% stenosis)
494010	male	63	no	yes	yes	no	no	Non-STEMI	LAD(70% stenosis)+LCX(85% stenosis)
465797	male	63	yes	no	yes	no	no	Non-STEMI	LCX(90% stenosis)
468921	male	56	yes	no	yes	no	no	STEMI	LAD(95% stenosis)
482408	male	60	yes	yes	no	no	no	STEMI	RCA(100% stenosis)
486690	male	42	yes	yes	yes	no	no	STEMI	RCA(95%-99% stenosis)
489735	male	60	yes	yes	yes	no	no	STEMI	LAD(99% stenosis)
